# Microstructure and Corrosion Characteristics of IN 625 Coating on Additively Manufactured 316L Stainless Steel in As-Fabricated Condition

**DOI:** 10.3390/ma19040812

**Published:** 2026-02-20

**Authors:** Prithwish Tarafder, Lingyin Meng, Tunji Adetayo Owoseni, Johan Moverare

**Affiliations:** 1 Department of Management & Engineering (IEI), Linköping University, SE-581 83 Linköping, Sweden; 2 Department of Physics, Chemistry & Biology (IFM), Linköping University, SE-581 83 Linköping, Sweden; 3 Department of Mechanical Engineering, Kwara State University, Malete 23431, Kwara, Nigeria

**Keywords:** 316L stainless steel, IN 625 coating, as-fabricated surface, electron beam powder bed fusion, HVAF

## Abstract

The microstructure and corrosion properties of electron beam powder bed fusion (EB-PBF)-fabricated 316L stainless steel are evaluated in the as-fabricated condition with and without the deposition of IN 625 coating. Different surface profiles were achieved by introducing layer thickness and a contour scan strategy as process variables. A potentiodynamic polarization test was used for corrosion testing, while state-of-the-art microstructural investigation techniques were employed to elucidate a possible link between the microstructure and corrosion properties of the samples. Results from this pilot study showed that the corrosion response was dictated by the combined effects of surface roughness, coating depth, coating morphology, and passive film characteristics. For specimens for which a contour scan strategy was not used, the coating hinted to an improved corrosion potential while it increased the corrosion rate for both layer thicknesses. On the contrary, for specimens where a contour scan was applied, as-fabricated samples trended towards better corrosion resistance than the coated samples. It is shown that the dross particles that are formed during EB-PBF processing influence the flattening mechanism of the coating, ultimately resulting in a coating deposit that is characterized by surface defects, microcracks, cavities, and incoherent splat boundaries.

## 1. Introduction

316L austenitic stainless steel (SS) is applied in chemical, petrochemical, and sea-water environments for its excellent corrosion properties. It is generally argued that the formation of a stable oxide surface layer causes the alloy to be corrosion-resistant; however, the nature of a corrosive medium may still impart damage to this protective layer, especially in the presence of chloride ions [1,2]. Furthermore, while cast and wrought 316L SS shows microstructural homogeneity, additively manufactured 316L SS shows a hierarchical and micro-segregation-rich microstructure. Alongside the effect of grain size reduction, which negatively influences pitting kinetics [3], factors such as molybdenum segregation at the cellular sub-grain boundaries, dislocation arrangement, process-induced defects like lack of fusion, grain texture, residual stress, and chromium- or manganese-rich nano-inclusions have been reported to alter the corrosion behavior of additively manufactured 316L SS, albeit these findings are limited to laser powder bed fusion (L-PBF) studies [4,5,6,7,8,9].

Typically, resistance to corrosion, erosion, or wear are enhanced in two ways: either by the addition of a surface coating material, or by creating an intrinsic protective layer using surface modification techniques such as nitriding, carburizing, etc. [1]. Prior research works conducted on the effect of coating material addition and the types of coating processes involving 316L SS demonstrate a mix of enhanced pitting resistance, improved corrosion potential, and varied corrosion rate responses. A range of coating materials such as copper [10], micro-scale vanadium [11], titanium nitride [12], alumina [13,14], hybrid silane reinforced with nano-scale zinc oxide [15], a combination of hydroxyapatite–titanium–zirconium [16], etc., have been reported in the literature. Coating processes also vary, including friction surfacing [17], atmospheric plasma spraying [13,14,16], shrouded plasma spraying [18], cold spraying [19], thermal spraying [20], etc.

While lightly reported for 316L SS [21], Inconel 625 is another popular choice of coating material investigated for other steel grades for better corrosion and wear properties [20,22,23,24,25,26,27]. IN 625 is a nickel–chromium–molybdenum–niobium-based superalloy known for its exceptional hot-corrosion resistance, high-temperature mechanical properties, improved fatigue properties under a corrosive medium, etc., making it an ideal coating material for improved operational efficiency in a demanding work environment. Nevertheless, to the best of our knowledge, no research work has directly dealt with IN 625 coatings on additively manufactured 316L SS. Furthermore, due to the high process-inherited surface roughness in additive manufacturing (AM), the components are typically ground or machined before the coating process to ensure proper bonding between the coating material and the substrate. While the effect of surface roughness on the corrosion properties indicates an inverse relationship of corrosion rate and corrosion potential [28,29,30,31,32], a rough interfacial profile has been proven to produce better adhesion properties [27,33]. Electron beam powder bed fusion (EB-PBF), another type of PBF-based AM technology, typically results in a poorer surface finish than L-PBF in as-fabricated conditions due to the feedstock powder particle size distribution and process kinetics. Hence, leveraging the aspect of surface roughness in EB-PBF processed 316L SS in the as-fabricated condition poses an interesting question as to what changes in the corrosion property are to be expected when it is coated with IN 625. In this exploratory pilot study, we thus investigated the possible microstructure–corrosion property linkage using different microstructural characterization techniques and the potentiodynamic polarization test method. Results suggest that both unpolished surface roughness due to the EB-PBF process and coating deposition influence the corrosion resistance of the tested samples. While a statistical inference of the results is not made in this study due to the sample size limitation, initial observations indicate that the coating improved the corrosion potential in low surface roughness accompanied with the smaller layer thickness condition.

## 2. Materials and Methods

### 2.1. Cuboid Sample Fabrication via EB-PBF

Cuboid specimens from feedstock 316L SS powder provided by Carpenter AB (Torshälla, Sweden with an actual composition of 17% Cr, 12% Ni, 2.3% Mo, 0.7% Si, 0.95% Mn, Fe balance, all in weight percent) were manufactured using the EB-PBF process via a Freemelt ONE^®^ machine (Freemelt, Göteborg, Sweden). The nominal powder size distribution ranged between 53 and 106 µm, with mostly spherical powder particles, occasionally attached with satellite particles. To examine the effect of the as-fabricated surface roughness, two variables were introduced: different layer thicknesses (LTs) of 70 µm and 105 µm using the same process parameters and a contour scan for selected samples with 0.5 mm offset from the geometry. Standard bi-directional raster scanning parameters were used to melt the bulk of the material (beam power of 660 W, beam scanning speed of 1700 mm/s, hatch spacing of 0.12 mm, and scan rotation of 90° between successive layers), while the samples selected with the contour scan strategy were fabricated with a contour scan with a beam speed of 2000 mm/s while keeping the other process parameters the same. A detailed representation of the powder and preheating conditions during the build job can be found in our previous research work using the same powder batch [34].

### 2.2. Coating and Microstructure Characterization Method

**Figure 1:**
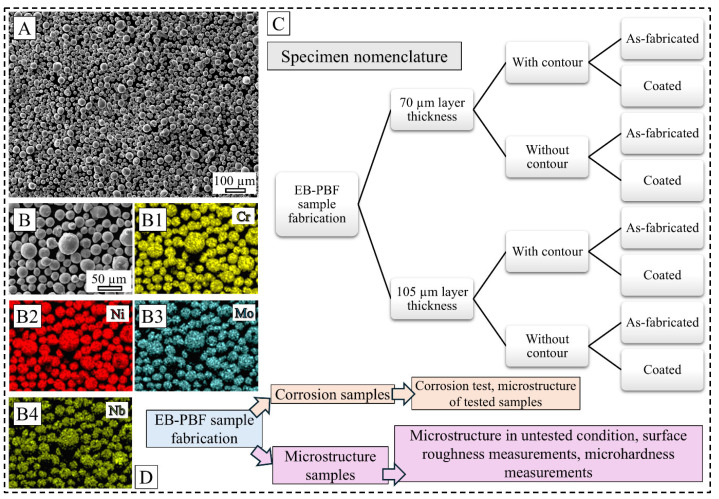
IN 625 feedstock powder characterization (**A**–**B4**); overall schematic of the specimen nomenclature used in the study (**C**); schematic of the overall workflow (**D**). Note different scale bars.

IN 625 powder (nominal composition of 20–23% Cr, 8–10% Mo, 3–4% Nb and Ta, max. 5% Fe, Ni balance, all in weight percent as per ASTM standard) was coated onto the as-fabricated unpolished surface of selected 316L SS samples via the high-velocity air fuel (HVAF) technique that employs a supersonic jet to accelerate the powder particles to achieve superior interlocking between the substrate and the coating material. Parameters utilized for the coating process are presented in Table 1. A scanning electron microscope (SEM) image (Figure 1A) of the feedstock coating powder demonstrates uniform spherical particles, with the primary elemental constituents of nickel, chromium, molybdenum, and niobium, as evidenced by the energy dispersive spectroscopy (EDS) image presented in Figure 1(B1–B4). Characterization of microstructural features in the samples in both as-fabricated and coated conditions was carried out using optical microscopy (OM), SEM, EDS, and electron backscatter diffraction (EBSD) techniques following the standard mechanical sample preparation steps. SEM and related works were conducted in a JEOL JSM-IT 500 (Tokyo, Japan) and Hitachi SU-70 (Tokyo, Japan) microscopes. Microhardness of the coated samples across the coating–substrate interface was recorded in a Struers DuraScan automated Vickers microhardness tester (Ballerup, Denmark) by taking at least 30 indentations in each region. The surface roughness was measured optically using a Leica DM6 OM (Wetzlar, Germany) and the Leica Map DCM software (version 8.2.10392) while considering the varying focal planes related to the surface profile, as per the ISO 4287 standard [35]. Each sample surface was imaged in five different areas, yielding 30 measurements per sample to offset the effect of any local surface defects.

### 2.3. Corrosion Test

For the corrosion test, epoxy-resin-mounted samples with a 1 cm^2^ open area along the build direction was exposed to a 3.5 wt.% NaCl aqueous solution at room temperature in a standard three-electrode cell setup. While the sample serves as the working electrode, a saturated Ag/AgCl works as the reference electrode and a platinum plate is inserted as a counter electrode. All samples were sonicated in isopropanol for three minutes prior to initial open circuit potential testing for three hours to eliminate the effect of external debris. Afterwards, electrochemical impedance spectroscopy (EIS) was conducted over the range of 100 kHz to 0.1 Hz with 10 mV of amplitude to evaluate the passive film properties, while the potentiodynamic polarization (PDP) test was realized at a scan rate of 1 mV/s from −0.1 V vs. the open circuit potential until the current reached 3 mA/cm^2^. The workflow and sample nomenclature utilized in this study is presented in Figure 1C,D. Electrochemical measurements were performed using an Autolab PGSTAT30 (Metrohm, Utrecht, Netherlands) associated with an FRA module. It is to be noted that only one sample per condition was tested in this study due to sample limitations.

## 3. Results and Discussion

### 3.1. Microstructure Characteristics

**Figure 2:**
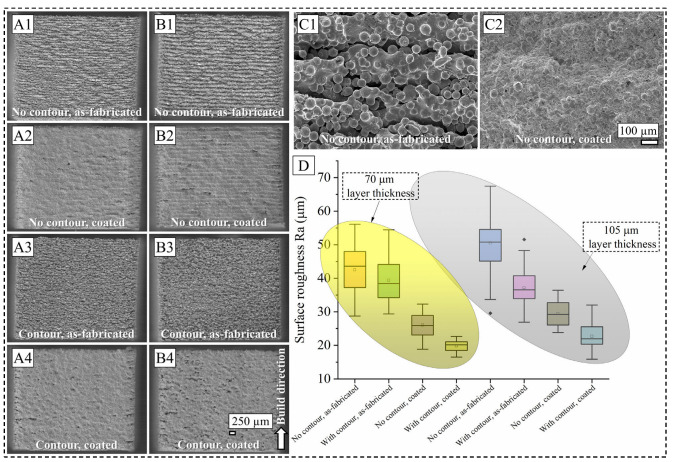
Overall surface OM image of 70 µm (**A1**–**A4**) and 105 µm (**B1**–**B4**) LT samples; SEM images of surface under as-fabricated and coated conditions for samples printed with 70 µm LT without contour (**C1** and **C2**, respectively); Ra surface roughness plot for all eight surface conditions (**D**). Note different scale bars.

Representative OM surface images from all eight surface conditions are presented in Figure 2. With the same process parameters, the volumetric energy density will be lower for a higher LT, hence leading to a risk of insufficient melting conditions. As seen in Figure 2(A1–A4), cuboids fabricated with a 70 µm LT show typical surface features characterized by layer fusion lines, voids, and partially melted powder particles. However, for cuboids fabricated with the contour scan strategy, the obvious appearance of a layered surface is diminished, and a rather homogeneous surface is produced. A similar trend is observed for the cuboids fabricated with a 105 µm LT, as shown in Figure 2(B1–B4). Due to the pronounced convoluted effect from the LT and un-melted and/or partially melted powder particles, all fabricated cuboids present with localized troughs. This surface morphology effect is manifested in the subsequent coating where the cuboids fabricated with a 105 µm LT presented more discontinuous coating than the 70 µm LT ones, represented by higher-contrast cavity regions. A closer look at the surface morphology under the as-fabricated condition (Figure 2(C1)) reveals the presence of cling-on surface features typical of the PBF process where high cooling rates, meltpool dynamics, interaction between the meltpool and surrounding powder particles lead to un-melted and/or partially melted powder particles getting attached to the surface contour. It is important to note that while the outer surface seems porous due to the process kinetics mentioned above, the inner core of the manufactured cuboids had relative densities greater than 99.5% for the process parameters used [34]. For the same process condition, when the sample is coated with IN 625, a continuous coating is obtained, as observed in Figure 2(C2). While some partially melted coating powder material is still seen on the coated surface, for the 70 µm LT, the coating material seems to have covered the crevices and troughs of the bulk surface.

**Figure 3:**
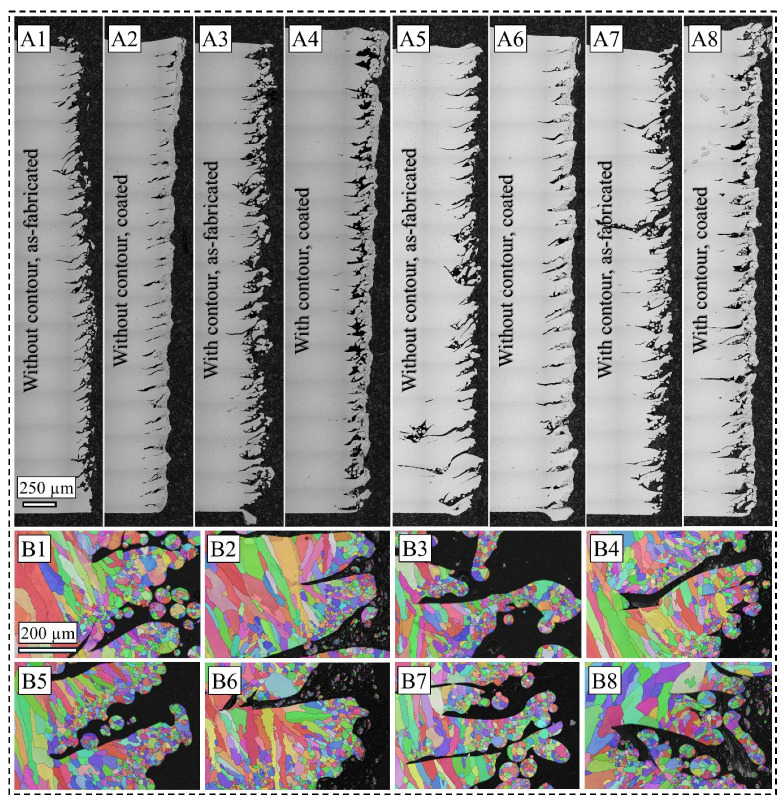
OM cross-sectional images of the surface along the build direction for all eight sample conditions ((**A1**–**A4**) = 70 µm LT; (**A5**–**A8**) = 105 µm LT). Note the label on each image for sample terminology. EBSD inverse pole figure maps (color mapped parallel to build direction) of as-fabricated samples without contour, coated samples without contour, as-fabricated samples with contour, and coated samples with contour, respectively, for 70 µm LT (**B1**–**B4**) and 105 µm LT (**B5**–**B8**). Note different scale bars.

To quantitatively assess the surface features in all eight sample conditions, the surface roughness measurements carried out using the optical technique are presented in Figure 2D. Ra analysis, a commonly used surface roughness metric in the EB-PBF literature [36,37] which describes the arithmetic mean deviation of the peak heights from the mean surface centerline profile, shows that the 70 µm LT samples have a lower surface roughness compared to the 105 µm LT samples. This is in accordance with the OM images and the process physics where meltpool capillary instabilities can occur due to insufficient melting of powder particles. Such melting phenomenon can drive balling and splashing of molten metals that collectively exacerbate the surface features, such as the dross particle formation [38,39]. Among different surface conditions, it is further noted that the coated samples have improved surface roughness compared to their as-fabricated counterparts, as expected from the HVAF process that achieves good interlocking between the coating material and the substrate. Furthermore, it is indicated that the samples manufactured with a contour scan have improved surface roughness than the samples without a contour scan, across two LTs and the coated–uncoated condition. It is surmised that the contour scan, which took place immediately after bulk melting, might have led to a coalescence effect of the surrounding powder particles near the surface. Similar findings have been reported in the L-PBF literature where the use of a contour scan was proven beneficial for surface finish improvement [40,41]. It should also be noted that Ra was chosen instead of any other potential metric such as Sa to be able to compare the global surface roughness with the cross-sectional 2D profiles presented in Figure 3.

To further examine the as-fabricated and coated surfaces, OM images of the cross-section along the build direction are presented in Figure 3(A1–A8). For samples fabricated with the 70 µm LT, the surface flaws are periodic, in line with the LT and the median powder particle size. However, the cross-sectional image of the sample fabricated with the contour scan reveals a more tortuous network of dross and partially fused particles. It is believed that the meltpool depth during the contour scan is lower than the surrounding powder particles, leading to insufficient melting and the wetting of turbulent liquid metal flow. Subsequently, the efficiency of the coating process depended on the prior surface profiles which affected the interaction between the impinging IN 625 powder particles and the 316L SS substrate surface. It is often argued that surfaces with a dense network of features presenting with frequent changes in the slope leads to the Cassie–Baxter state of wetting behavior, while a Wenzel wetting is achieved in cases where the wetting contact angle is <90°. Due to the surface roughness created by the EB-PBF process, the coating material may either flow over the surface profile and create a flattening effect, i.e., a splat, or can get pinned by the depression or valleys [33]. Furthermore, low fluidity and formability [25] of partially or un-melted 316L SS powder particles leads to inconsistent plastic deformation of the coating particles upon impingement. Consequently, this mechanism imposes a dominating effect on the subsequent corrosion properties of the samples where the consistency and morphological features of the coating film play a significant role. For instance, the IN 625 coating film sometimes seems to cover the average of the surface profile, as shown in Figure 3(A2), while sometimes it creates coating accumulation around the peak near a depression wherein the coating particles do not penetrate through the valley, as shown in Figure 3(A8).

From another microstructural perspective, the near-surface grain size and grain boundary fraction are reported to influence the corrosion properties of several material systems [3,5,9,26,42]. Typically, sub-grains associated with a high fraction of low-angle grain boundary and small surface grains associated with high grain boundary fraction create abundant diffusion paths for the metallic elements across the substrate–coating interface. Moreover, due to the inherent high cooling rate scenarios of both the EB-PBF process [34] and thermal spraying techniques [24], the solid solution diffusion of heavier elements such as molybdenum and niobium becomes unfavorable in an α-Ni matrix, and they tend to precipitate along the sub-grain boundaries. Although the sub-grains are not apparently observed for the scan step size used in the EBSD acquisition, the high fraction of grain boundaries due to small surface grains is readily discernible for all eight surface conditions, as presented in Figure 3(B1–B8). As masking the near-surface grains for quantitative grain size measurements can be a subjective annotation, a qualitative assessment of the grain morphology reveals smaller surface grains for the 105 µm LT samples (Figure 3(B5–B8)) than the 70 µm LT samples (Figure 3(B1–B4)), resulting from more severe cooling rate and thermal gradient kinetics due to lower volumetric energy density. This observation is supported by the higher fraction of high-angle grain boundaries present in the 105 µm LT samples, as measured from EBSD data.

**Figure 4:**
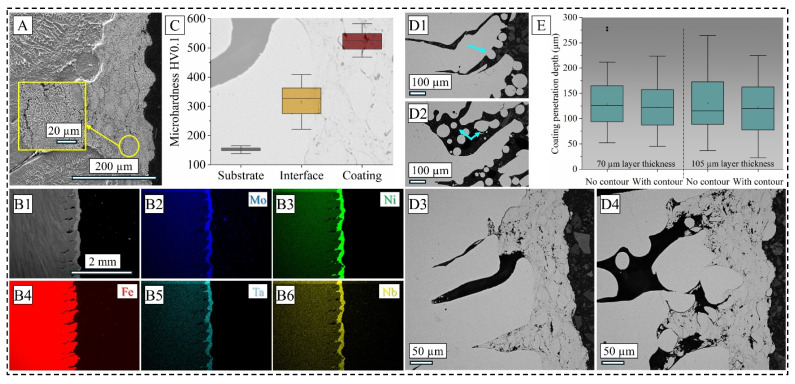
Etched microstructure SEM image of a representative sample (**A**); EDS map of the coating cross-section along the build direction (**B1**–**B6**); microhardness map spanning the substrate-interface-coating region (**C**); OM images of as-fabricated samples and coated samples without contour (**D1** and **D3**, respectively) and with contour (**D2** and **D4**, respectively) for 70 µm LT; penetration depth of coated samples (**E**). Note different scale bars.

Further microstructural characterization after chemical etching, as shown in Figure 4A, demonstrates a typical AM-fabricated cellular dendritic grain morphology of the substrate, while the coating presents with several microcracks and higher contrast phases, mostly oxides formed during the process. It is commonly reported for the IN 625 coating where Cr_2_O_3_ is formed on the film surface with a local chromium depletion [24,25]. However, in the low magnification EDS map presented in Figure 4(B1–B6), only major elements such as molybdenum, niobium, nickel, and tantalum were found in the coating region with high contrast, while chromium was more uniformly distributed between the substrate and the coating material.

Figure 4C illustrates the microhardness response from representative samples shown in the foreground of an OM image of the interface for a pictorial representation. As expected, the 316L SS substrate registered an average Vickers microhardness of 152 HV0.1, whereas the coating region of IN 625 recorded an average of 522 HV0.1, with the interface region falling in between with an average of 313 HV0.1. This multiple-fold increase in hardness in IN 625 is mainly attributed to the strengthening precipitate phases of molybdenum and niobium, which has been reported before in [22,23], and alludes to an improved wear resistance of coated 316L SS.

Nevertheless, the aspect of an inherent surface roughness and subsequent coating adhesion can sufficiently modify corrosion properties and hence needs to be considered in further detail. As seen in Figure 4(D1–D4), dross particles are found abundantly in the as-fabricated cuboid surface (D1 and D2), as shown with the light blue arrows. Furthermore, while the OM collage images of the surface presented in Figure 2 alluded to more uniformity in the contoured sample, the cross-sectional image in Figure 4(D2) shows a more sporadic arrangement of un-melted powder particles and the surface-connected dross formation. For the sample fabricated without contour (shown in Figure 4(D3)), the coating seemed to have penetrated the crevices of the virgin surface where the wetting condition was favorable for material flow. However, such flow is severely restricted for the contoured sample, as shown in Figure 4(D4), where splats are formed along with voids and cracks due to the rupture pressure of two adjoining splats over the peaks of the surrounding valleys. This necessitated quantification of the penetration depth of the coating across all coated conditions, which signifies the actual coating depth considering the prior surface profile, and the result is presented in Figure 4E. As expected from the OM cross-sectional image illustrated in Figure 3, the no-contour condition in the 70 µm LT showed the highest average depth of 133 µm, whereas the penetration depth decreased as the LT increased. For contour strategy, it is seen that for both LTs, the average penetration depth of the coating is smaller than their no-contour counterparts, counterintuitive from their surface roughness relationship. However, it should be noted that the roughness was measured via optical profilometry, a technique that relies on light diffraction and contrast based on feature height. Hence, it is believed that the contour scans which create more turbulent meltpool flows and stronger particle splashing or recoil reveal its true significance when cross-sectional images are taken. This effect is partially masked while measuring the surface roughness of the bulk sample in a non-destructive way, where the coalescence effect of the surrounding powder particles dominates. Nevertheless, this difference in the penetration depth is expected to influence the corrosion properties of respective surface conditions, which is described in detail in the next section.

### 3.2. Corrosion Properties

**Figure 5:**
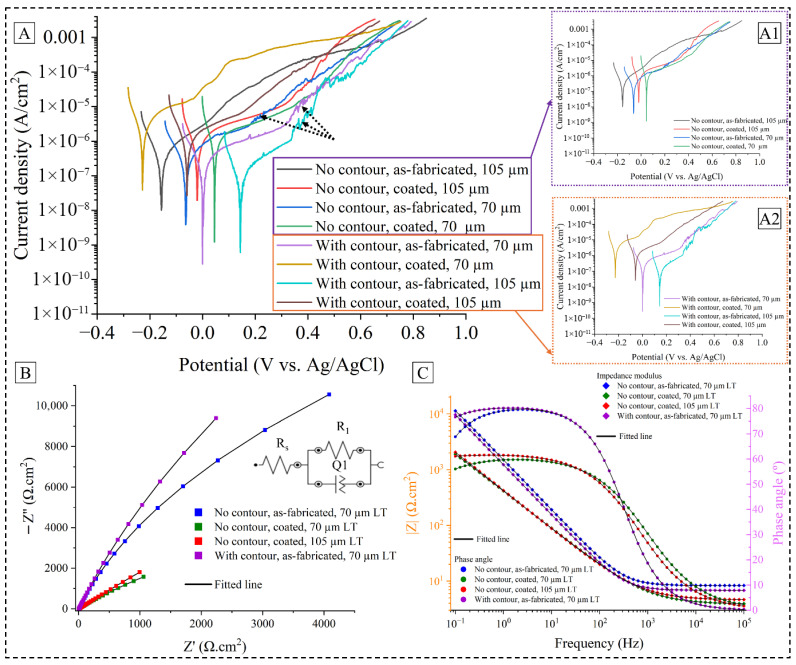
Potentiodynamic polarization curves for all surface conditions (**A**); zoomed-in PDP for samples without (**A1**) and with (**A2**) the contour scan; Nyquist plot for selected surface conditions along with the equivalent electrical circuit shown in the inset (**B**). Bode impedance and phase angle plot from EIS data (**C**).

From the microstructural differences among different coated and uncoated surface conditions, it is expected that these features will impact the respective corrosion properties. Results from the corrosion tests are presented in Figure 5A, which shows the polarization curves along with the calculated E_corr_ and I_corr_ values from Tafel extrapolation, as shown in Table 2. In general, it is seen that the EB-PBF-processed as-fabricated samples show a superior corrosion resistance than the L-PBF-processed 316L SS [42,43,44] under a similar electrochemical setup, while a few variants in L-PBF studies demonstrated marginally better corrosion resistance [6,8]. Between the two process technologies, the thermal history of the process and the solidification kinetics invariably lead to a much different microstructure wherein L-PBF-processed 316L SS samples are generally characterized with high dislocation density, finer grain size, and cellular sub-structure with elemental micro-segregation, all of which contribute to inferior corrosion properties. However, focusing on the present study, the coated samples without a contour scan showed a shift toward more positive E_corr_, indicating a possible nobler corrosion potential state than the as-fabricated samples. Between the two LTs, it is further revealed that the 70 µm LT samples have better corrosion protection compared to the 105 µm LT samples. However, with the contour scan, it is seen that the as-fabricated samples present with nobler corrosion characteristics than the coated ones, shifting towards positive potential. Furthermore, for this process condition, the 105 µm LT samples showed better corrosion properties than the 70 µm LT samples.

These two different trends align well with the microstructural observation presented in the previous section, where it is shown that the coating achieved a better surface finish and a deeper penetration of coating film for the 70 µm LT than the 105 µm LT when no contour scan was applied. However, this trend is reversed when contour scan is applied, resulting in the opposite outcome in the coating penetration and subsequent response to the corrosive medium. It is believed that the higher LT has led to a dominating staircase effect, which is seen in the OM image presented in Figure 2. Such wider arrangements of protrusion and depression in the surface profile helps with the flow of the coating material due to its smaller change in the slope as compared to the lower LT samples. Furthermore, powder particles partially melted during the bulk scan achieve a favorable meltpool volume during the contour scan to recoil back into the surface centerline, making the coating penetrate deeper into the substrate surface. This line of reasoning is also applicable to the as-fabricated condition that suggests a similar trend of better corrosion resistance in the 105 µm LT samples than the 70 µm LT ones fabricated with the contour scan, as shown in Table 2.

To decipher the effect of the process-related variables, i.e., LT and contour scan, PDP data are reproduced for the samples without a contour scan in Figure 5(A1) and the samples with a contour scan in Figure 5(A2). In the case of the samples fabricated without a contour scan, it is seen that the coating increased the corrosion rate with higher I_corr_ values, albeit improving the corrosion potential. This observation is in line with several studies where coated specimens show incremental anodic reactions when a slight overpotential is applied [10,17,20]. However, with a contour scan, both corrosion potential and corrosion rate worsen with the application of coating material, indicating a combined deleterious effect from coating discontinuity, surface flaws, lower penetration depth, etc., for the tested samples. Furthermore, metastable pitting is observed in the as-fabricated samples for both LT samples, as suggested by the current density spikes, indicated by the black dashed arrows. However, the onset of pitting varies among the surface conditions, dictated by the initial passive film on the sample and the rate of dissolution of the metal due to damage in the passive film. To better understand the nature of passive film and its response to corrosion attack, EIS data of selected specimens are fitted for Nyquist and Bode plots. Figure 5B exhibits the Nyquist plot for four sample conditions, viz., as-fabricated without contour (70 µm), coated without contour (70 µm), coated without contour (105 µm), and as-fabricated with contour (70 µm). Among these surface conditions, it is possible to infer the interwoven trends of contour scan strategy, LT, and coating material. As the arc length in a Nyquist plot represents the barrier properties of the passive film [44,45,46], it is interpreted that for the as-fabricated condition, the samples with a contour scan are better protected against corrosion compared to the samples without a contour scan, as is also evident from Table 2 data. Moreover, for the scenarios without contour scan, the as-fabricated samples possess better passivation mechanism than the coated samples, which manifests into reduced corrosion density, in line with the observations from the PDP test.

Bode plots of impedance and phase angle bear a significant understanding of the passive film formation and retention. While increasing the absolute value of impedance generally refers to a better corrosion resistance, the behavior changes from resistance to capacitance as the phase angle increases from zero to 90 degrees. However, as seen in Figure 5C, all samples were below 90 degrees within the frequency used in the EIS measurement, which indicates a non-ideal capacitive behavior of the passive film, presumably affected by the respective microstructural features such as coating penetration depth, continuity of coating film, microcracks, etc. Both as-fabricated tested samples were closer to 90 degrees, corroborating their superior corrosion resistance than the coated samples. Similarly, the higher impedance values of the as-fabricated samples within the intermediate frequency range signify that the passive film of these samples has a higher polarization resistance and thus a better corrosion resistance [42,44,45].

In accordance with the interpretation of the obtained EIS results, an equivalent electrical circuit (EEC) is proposed and fitted with the test data against a threshold chi-squared value, as shown in the inset of Figure 5B. The simulation model employed an EEC with a solution resistance R_s_, a constant phase element Q_1_ and a resistance R_1_ in parallel. While R_1_ signifies the influence of the passive oxide film kinetics in corrosion protection, the non-ideal capacitance behavior is encapsulated by the Q_1_ metric [45], which in turn is represented by the pseudo-capacitance denoted by *Y* and a migration coefficient symbolized by *n* [46]. Table 3 shows these EEC parameters for the selected surface conditions. Again, it shows a sharp increase in the R_1_ value that suggests an increase in the oxide film protection when a contour strategy was used in the as-fabricated condition. When the coating is applied for the same LT, it is seen that the R_1_ value substantially declines along with an increase in *Y* and a decrease in *n*, which further illustrates the weakening nature of the barrier protection offered by the passive film. It is possible that such behavior is accompanied by the diffusion of electrochemical mass transport through the microcracks and splat boundaries in coating that provide the required opening for the corrosion medium to penetrate through the coating thickness. Similar effects can be ascribed to the differences observed between coated samples without a contour scan for two LTs, wherein the 105 µm LT sample showed a reduced corrosion rate, as suggested by an increase in R_1_ and a decline in *Y*, compared to the 70 µm LT sample. However, the improved corrosion potential towards a more noble state for the 70 µm LT sample proposes a convoluted effect of the penetration depth and morphology of the coating layer on the corrosion behavior, which necessitate detailed microstructural characterization of the surface after the corrosion test.

**Figure 6:**
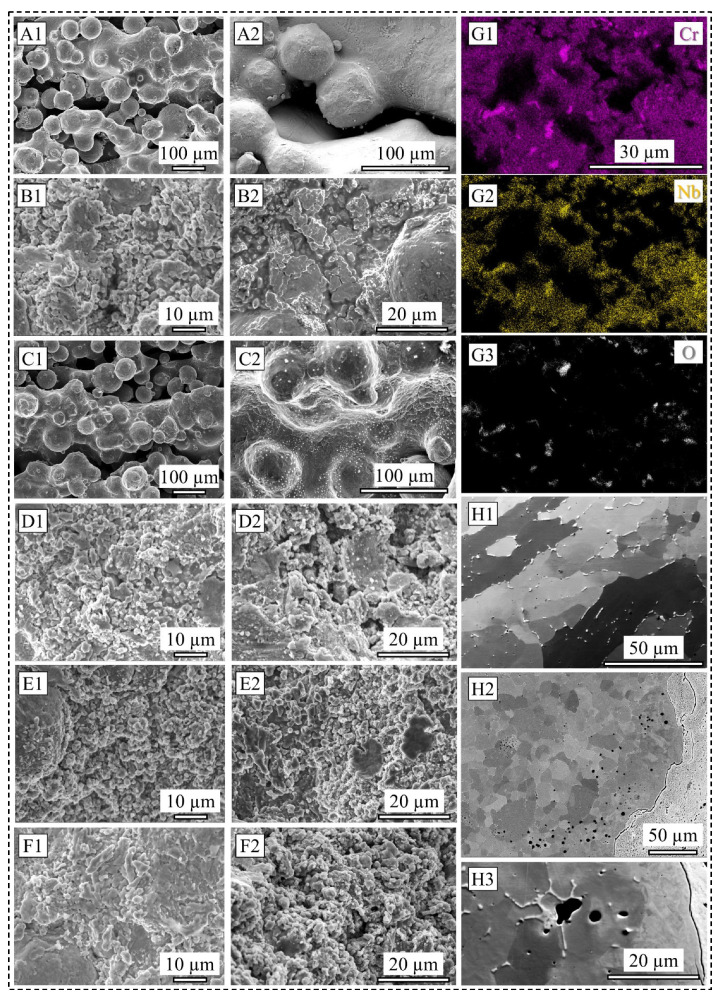
SEM images of surface before and after corrosion test for 70 µm LT as-fabricated sample without contour (**A1**,**A2**); 70 µm LT coated sample without contour (**B1**,**B2**); 105 µm LT as-fabricated sample without contour (**C1**,**C2**); 105 µm LT coated sample without contour (**D1**,**D2**); 70 µm LT coated sample with contour (**E1**,**E2**); and 105 µm LT coated sample with contour (**F1**,**F2**); EDS map of a representative surface showing the elemental distribution of chromium, niobium, and oxygen (**G1**–**G3**); pitting in 70 µm LT coated sample without contour (**H1**) and 105 µm LT coated sample without contour (**H2**,**H3**). Note different scale bars.

Figure 6(A1–F2) display a collection of SEM images of several surface conditions before and after the corrosion test to elucidate the effect of corrosion. The condition of the as-fabricated surface without a contour scan of 70 µm LT is presented in Figure 6(A1,A2), before and after the corrosion test, respectively. It is seen that corrosion test did not alter much of the surface morphology, except for certain small micrometer-scale oxide particles sporadically found around the partially melted 316L SS powder particles. The corroded surface of the same condition for the 105 µm LT presented in Figure 6(C1,C2), however, suggests observable differences, as identified by numerous corrosion product particles precipitated onto the surface. This signifies the increased corrosion rate and a lower corrosion potential, as found for the 105 µm LT sample, compared to its 70 µm LT counterpart. All coated surface conditions are presented in Figure 6(B1,B2) (without contour, 70 µm LT), Figure 6(D1,D2) (without contour, 105 µm LT), Figure 6(E1,E2) (with contour, 70 µm LT), and Figure 6(F1,F2) (with contour, 105 µm LT). In general, when the corrosion rate, i.e., the damage to the protective passive oxide film, is high, the corroded surface reveals micrometer-scale irregularly shaped particles. Furthermore, features such as fragmentation of the coating splat, progression of microcracks, and cohesive failure caused by spallation are observable in the surface conditions that led to a reduced corrosion potential and an increased corrosion rate, thereby demonstrating lower corrosion resistance in those tested samples.

Characterization of the higher contrast particles on the corroded surface reveals the presence of chromium, niobium, and oxygen, as shown in Figure 6(G1–G3). This elemental distribution suggests the presence of commonly found Cr_2_O_3_ and oxide variants of niobium. Although previous studies have shown that such oxides improve the corrosion properties of coated specimens [7,24,25], it is also important to consider the morphology and the consistency of the protective barrier. Non-uniform distribution of such oxide films, when found at sufficient length scales, can negatively impact the corrosion performance of the material [8,9,27,30]. Furthermore, it is widely accepted that internal cracks and pores within the coating material may deleteriously affect the breakdown potential, which is manifested in the combined effect of the LT and contour scan strategy that result in the surface-connected and layered cavities.

Figure 6(H1–H3) demonstrates stable pitting phenomena in the coated specimens fabricated without a contour scan for two LTs. While metastable pitting is observed in the PDP curves for the as-fabricated condition, stable pitting is postulated to be driven by the coalescence and growth of merging pits after the barrier potential is surpassed. It is frequently reported that AM-processed 316L SS shows more propensity towards a stable pit growth than the wrought counterpart once pitting is initiated. This is mostly revealed through localized corrosion accompanied by an increased pitting frequency and a reduced current density at the pitting potential [4,8,30]. For the 105 µm LT sample (shown in Figure 6(H2,H3)), pitting seems more clustered towards the interface area than for the 70 µm LT sample (presented in Figure 6(H1)). The magnified view in Figure 6(H3) reveals such merged pitting around the molybdenum–chromium-rich phase observed at the grain boundary. It is likely that such elemental segregation along with porosities may have degraded the passivity of the protective film and may have nucleated pits at the overpotential range by activating a combination of micro-galvanic and crevice corrosion modes [4,8]. The growth of this pitting was further escalated due to progressive breakdown in passivation, thus steadily increasing the corrosion rate as the potential went beyond the pitting potential.

It is therefore deduced that the corrosion performance of the chosen surface conditions in this exploratory study is possibly influenced by a synergistic effect of 316L SS passive film integrity, IN 625 coating consistency, coating penetration depth, and the overall microstructure near the interface. However, it is acknowledged that a single test specimen per condition arising from the fabricated sample limitation from the mentioned powder batch severely restrains us from making a statistically conclusive remark. It is also known that the electrochemical tests are sensitive to surface integrity and localized features; hence, relying on a single data point only allows us to make observational correlations. Furthermore, optimization of the contour scan strategy or the coating process parameters may impart different correlational outcomes of the corrosion response of the surface conditions. While it is commonly reported that surface flaws below 50 µm do not contribute to a decline in corrosion resistance [5,8,9,44], severe surface defects such as lack of fusion, large un-melted powder particles, etc., due to unoptimized process conditions can still influence the corrosion properties as they increase the surface area subjected to a corrosive medium. Nevertheless, this pilot study, directed towards understanding the IN 625 coating performance on EB-PBF-processed 316L SS, provides preliminary insights into the key parameters that can influence corrosion properties. Importantly, it shows the feasibility of depositing a coating material on an unpolished as-fabricated surface while still retaining good balance of microstructural details and corrosion resistance, making it industrially relevant to delve into more dedicated studies involving a statistically significant number of samples.

## 4. Conclusions

This experimental methodological study attempted to understand a possible link between the microstructure and corrosion properties of EB-PBF-processed 316L SS coated with IN 625 in the as-fabricated unpolished condition. It is observed that the surface roughness stemming from the processing conditions plays a significant role in the compactness and robustness of the coating film. Furthermore, results from the PDP tests suggest that for the surfaces manufactured without a contour scan, the application of coating leads to a more positive corrosion potential while also recording a higher corrosion rate. However, the opposite trend is found when a contour scan is applied during EB-PBF processing, demonstrating its importance in creating the surface profile and dominating the outcome of the coating process wherein the penetration depth of the coating material within and around the crevice and valleys of the fabricated surface is shown to be a significant metric. Furthermore, the influence of the LT is manifested in the obtained surface roughness and coating cohesiveness for the tested conditions. While lower LT led to a smoother surface finish in all surface conditions, higher LT with a contour scan delivered better corrosion properties, partly due to the ability to achieve higher coating penetration because of a wider staircasing effect. Meanwhile, from the PDP test, as-fabricated samples showed a metastable pitting behavior, typical of AM 316L SS; however, samples with coating implied more stabilized pitting either found sporadically or in clusters. In this regard, for no-contour conditions, lower LT seems to have led to lowered pitting corrosion, while the 105 µm LT showed more pitting instances, primarily driven by the breakdown of the passive film owing to the coating robustness, which is shown to be affected by the prior EB-PBF-processed surface roughness. However, considering that the study was conducted with one sample per condition, these observations are offered as probable trends as opposed to certain correlational links. These links can only be realized after a full-scale study aiming to achieve statistical thoroughness is conducted.

## Figures and Tables

**Table 1:** HVAF process parameters using an M3 spray torch with 4L4C nozzle.

Parameters	Values	Parameters	Values
**Air pressure**	106 psi	**Stand-off distance**	325 mm
**Fuel 1 pressure**	100 psi	**Traverse speed**	100 m/min
**Fuel 2 pressure**	110 psi	**Step size**	5 mm
**Carrier gas flow**	40 L/min	**Number of passes**	10
**Feed rate**	120 g/min	**Final thickness**	191 µm (expected)

**Table 2:** Calculated Ecorr and Icorr values from Tafel extrapolation using Nova 2.1 Metrohm Autolab software.

Surface Condition	E_corr_ (V vs. Ag/AgCl)	I_corr_ (µA/cm^2^)	LT
**As-fabricated samples without contour**	−0.061	0.152	70 µm
**Coated samples without contour**	0.046	0.438	70 µm
**As-fabricated samples with contour**	0.004	0.162	70 µm
**Coated samples with contour**	−0.228	1.197	70 µm
**As-fabricated samples without contour**	−0.154	0.198	105 µm
**Coated samples without contour**	−0.019	0.367	105 µm
**As-fabricated samples with contour**	0.145	0.043	105 µm
**Coated samples with contour**	−0.056	0.559	105 µm

**Table 3:** EEC parameters used for fitting the EIS data.

Surface Condition	Rs (Ω·cm^2^)	R_1_ (kΩ·cm^2^)	Q_1_		LT
			*Y* (µMho × s*^n^*/cm^2^)	*n*	
**As-fabricated without contour**	8.32	53.31	159	0.9	70 µm
**Coated without contour**	3.85	18.80	700	0.68	70 µm
**Coated without contour**	4.57	97.75	534	0.69	105 µm
**As-fabricated with contour**	6.81	124.81	186	0.9	70 µm

## Data Availability

The original contributions presented in this study are included in the article. Further inquiries can be directed to the corresponding author.
